# Fc receptor-like 1, 3, and 6 variants are associated with rheumatoid arthritis risk in the Chinese Han population

**DOI:** 10.1186/s41021-021-00213-2

**Published:** 2021-10-07

**Authors:** Yonghui Yang, Dandan Li, Chunjuan He, Linna Peng, Shishi Xing, Mei Bai, Hao Rong, Dongya Yuan, Yongjun He, Xue He, Li Wang, Tianbo Jin

**Affiliations:** 1 Clinical Laboratory, Xi’an 630 Hospital, Yanliang, Xi’an, Shaanxi China; 2 Key Laboratory of Molecular Mechanism and Intervention Research for Plateau Diseases of Tibet Autonomous Region, School of Medicine, Xizang Minzu University, #6 East Wenhui Road, Xianyang, 712082 Shaanxi China

**Keywords:** RA, *FCRL1*, *FCRL3*, *FCRL6*, Polymorphisms

## Abstract

**Background:**

Rheumatoid arthritis (RA) is the most common autoimmune system diseases in our world. More studies in recent years have shown that *FCRL* gene polymorphisms is closely related to autoimmune diseases. It is suggested that genetic factors play a crucial role in the pathogenesis of this disease. In this study, we aimed to investigate the relationship between *FCRL1* rs2050568, *FCRL3* rs2317230 and *FCRL6* rs58240276 polymorphisms and RA risk in the Chinese Han population. 506 with RA patients and 509 healthy controls were recruited in this study, and the single nucleotide polymorphisms (SNPs) was successfully genotyped using the Agena MassARRAY platform. Odds ratios (ORs) and 95% confidence intervals (95% CIs) after adjusting for age and gender were conducted to assess these SNPs polymorphisms and RA risk. The multifactor dimensionality reduction (MDR) method was conducted to analyze SNP-SNP interaction.

**Results:**

Our results revealed that there no significant association was observed between the allele and genotype frequencies among these SNPs and RA risk (all *p* > 0.05). Straified analysis by age and gender, the results confirmed that *FCRL1* rs2050568 T/T genotype enhanced the risk of RA in females (*p* = 0.014). The G/T - T/T genotype of *FCRL3* rs2317230 was correlated with a decreased RA risk in males (*p* = 0.021). We also observed that the C/T-T/T genotype of *FCRL6* rs58240276 was increased the risk of RA in the group at age >  54 years (*p* = 0.016). In addition, *FCRL1* rs2050568-TT, *FCRL6* rs58240276-TT and *FCRL1* rs2050568-TT, *FCRL3* rs2317230-TT, *FCRL6* rs58240276-TT are the best models for multi-site MDR analysis (*p* < 0.05), and the two best models mentioned above and classes RA have the most significant correlation.

**Conclusions:**

Our study demonstrated that *FCRL1* rs2050568, *FCRL3* rs2317230, and *FCRL6* rs58240276 polymorphisms were correlated with RA susceptibility in the Chinese Han population.

**Supplementary Information:**

The online version contains supplementary material available at 10.1186/s41021-021-00213-2.

## Introduction

Rheumatoid arthritis (RA) one of the most concerning inflammatory diseases with its frequency, chronicity, and system characterized by synovial destruction and joint inflammation, which leads to reduce the quality of life and even causes to the disability [1, 2]. It was estimated that the prevalence of RA was more than 0.8% in the our world [3]. Up to now, the etiologies of RA remain unknown. Recently, numerous epidemiologic studies confirmed that genetic factorsone of the two essential and fundamental factors that environment factor is the other, may play an important role in the occurrence and development of RA [4, 5]. Moreover, increasing genome-wide association studies (GWAS) have been identified more than hundreds of single-nucleotide polymorphisms (SNPs) correlated with autoimmune diseases, such as ankylosing spondylitis (AS), juvenile idiopathic arthritis (JIA), and RA [6–8].

Fc receptor-like (*FCRL*) gene family a new member of the Ig superfamily, which encodes the protein may play an essential role in regulating B signaling [9]. It is reported that the extracellular part of *FCRL* molecules contains multiples numbers of Ig-like domains and their cytoplasmictail contains immunoreceptor tyrosine-based activation motifs (ITAMs) and immunoreceptor tyrosine-based inhibitory motifs (ITIMs) [10]. In addition, the *FCRL* gene family contained six members with *FCRL1*, *FCRL2*, *FCRL3*, *FCRL4*, *FCRL5* and *FCRL6*. *FCRL1–5* was significantly expressed by B cells. However *FCRL6* was expressed by T cells and NK cells [11]. To date, the *FCRL* gene expression has been extensively assessed in human malignancies involving mantle cell lymphoma and multiple myeloma [12, 13]. Nowadays, increasing researches have been provided many evidences which demonstrated that *FCRL* gene polymorphisms, especially *FCRL3*, were associated with various autoimmune diseases including AS and RA [14–16]. Okada et al. [17] conducted a European-specific and Asian-specific GWAS meta-analysis by evaluating about 10 million SNPs, and found 42 new RA risk sites at the genome-wide significance level. Among them, the T/G genotype of *FCRL3* rs2317230 polymorphism significantly increased the risk of RA in the European and Asian populations (OR = 1.07, 95% CI = 1.04–1.10, *p* = 1.0E-05, OR = 1.10, 95%CI = 1.04–1.16, *p* = 3.1E-04, respectively). Therefore, the *FCRL3* rs2317230 polymorphism is associated with the risk of RA in the Europeans and Asians populations.

Through performed a case-control analysis comprising 506 patients with RA and 509 healthy controls in the Chinese Han population, our study is the first to elucidate the relationship between *FCRL1* rs2050568, *FCRL3* rs2317230, *FCRL6* rs58240276 polymorphisms and RA in the Chinese Han population.

## Material and methods

### Subjects

All individuals including 506 patients with RA and 509 healthy controls were enrolled from the Yanliang 630 hospital from the October 2016 to January 2019. All the individuals were unrelated Chinese Han people. In the current study, we performed the same exclusion criterion: any patients and healthy controls having cancer, transplantation or other autoimmune diseases were excluded. In addition, this study was conducted in accordance with the Declaration of Helsinki and the protocol was approved by the Ethics Committee of the Affiliated Hospital of Xizang Minzu University. In the same vein, written informed consents were obtained from all subjects.

### DNA extraction and genotyping

Five whole blood samples were available from each participants. According to the manufacturer’s instructions, genomic DNA was extracted from whole blood samples using the GoldMag-Mini Whole Blood Genomic DNA Purification Kit (GoldMag. Co. Ltd., Xi’an, China). The DNA concentration and purity were assessed using spectrophotometer (NanoDrop 2000; Thermo Fisher Scientific, Waltham, MA, USA). In this study, *FCRL1* rs2050568, *FCRL3*rs2317230, and *FCRL6* rs58240276 were chose to investigate the influence on the risk of RA from the 1000 Genomes Project (http://www.1000genomes.org/) with the minor allele frequency (MAF) > 5% [18]. The amplification and extension primers were performed through the Agena Bioscience Assay Design Suite V2.0 software (https://agenacx.com/online-tools/), following the guideline. Subsequently, SNPs genotyping and data analysis were conducted using the Agena MassARRAY platform (Agena Bioscience, San Diego, CA, USA) and Agena Bioscience TYPER version 4.0, respectively.

### Statistical analyses

SPSS 20.0 (SPSS, Chicago, IL, USA) software was used for statistical analysis [19]. For all subjects, the difference of age and gender was applied by the Pearson’s chi-square test and independent sample Student’s t-test. Among the healthy controls, the genotype frequencies were calculated to evaluate the departure from Hardy-Weinberg equilibrium (HWE) using the chi-square test. In addition, the relationship between these SNPs and RA risk was estimated with the values of odds ratios (ORs) and 95% confidence intervals (CIs) using the logistic regression analysis after adjusting for age and gender. The analysis was conducted by the PLINK 1.07 software [20]. All *p* values were two-sided, and *p* < 0.05 was considered to be statistically significant. In addition, we used multifactor dimensionality reduction (MDR, version 3.0.2) to evaluate the impact of SNP - SNP interaction on the risk of RA. This method can reveal the high-order interactions between genes related to a specific phenotype, thereby identifying multilocus genotype combinations which are related a high or low risk of disease. The *p* value analyzed using MDR software was calculated using the χ^2^ test.

## Results

### Characteristics of subjects

In the current study, 506 patients with RA (135 males and 371 females, mean age 54.35 ± 11.69 years) and 509 unrelated healthy controls (134 males and 375 females, mean age: 54.39 ± 12.02 years) were recruited. No significant difference in the distribution of gender between the patients and healthy controls was observed (*p* > 0.958). However, we found that the distribution of age between the two groups was significant difference (*p* = 0.038). Subsequently, we further analyzed the clinical parameters among 506 patients with RA. The mean ± SD of CRP, RF, ESR, and CCP were 31.05 ± 40.25 mg/L, 164.09 ± 147.21 KIU/L, 44.28 ± 30.86 mm/h and 75.11 ± 60.78 RU/ml, respectively. The characteristics of patients and healthy controls are summarized in Table 1.

**Table 1:** Characteristics of cases and controls

Variables	Cases (*n* = 506)	Controls (*n* = 509)	*p*
Age, years (mean ± SD)	59.80 ± 9.08	59.80 ± 10.63	0.038^*^
≤ 54	262 (51.78%)	260 (51.08%)	
> 54	244 (48.22%)	249 (48.92%)	
Gender			0.958
Male	135 (26.68%)	134 (26.33%)	
Female	371 (73.32%)	375 (73.67%)	
Clinical parameters			
CRP (mg/L)	31.05 ± 40.25		
RF (KIU/L)	164.09 ± 147.21		
ESR (mm/h)	44.28 ± 30.86		
CCP (RU/ml)	75.11 ± 60.78		

SD: standard deviation; RA: rheumatoid arthritis; SD: standard deviation; CRP: C-reaction protein; RF: rheumatoid factor; ESR: erythrocyte sedimentation rate; CCP: anti-cyclic citrullinated peptide

*p* values were calculated from χ^2^ test

*p < 0.05 indicates statistical significance

### Association between SNPs and RA risk

The detailed information including SNP ID, position, gene, allele, MAF, and HWE *p*-value is displayed in Table 2. Our results suggested that the genotype distribution of all candidate SNPs was in accordance with HWE among the healthy controls group (all *p* > 0.05). Furthermore, the MAF of these SNPs was not significantly associated with RA risk in the Chinese Han population (*p* > 0.05). In order to further evaluate the relationship between *FCRL1* rs2050568, *FCRL3* rs2317230, *FCRL6* rs58240276 polymorphisms and RA risk, three different genetic models such as the dominant, the recessive and the log-additive models after adjusting for age and gender were performed. Nevertheless, no significant correlation between the three SNPs polymorphisms and the risk of RA was observed in this study (Table 3**)**.

**Table 2:** Basic characteristics and allele frequencies among these SNPs

SNP	Gene	Chr	Allele	MAF		HWE *p*–Value	OR(95% CI)	*p*
				Case	Control			
rs2050568	*FCRL1*	1	T/C	0.23	0.20	0.891	1.17(0.94–1.44)	0.155
rs2317230	*FCRL3*	1	T/G	0.37	0.37	0.924	1.03 (0.86–1.23)	0.775
rs58240276	*FCRL6*	1	T/C	0.23	0.20	0.891	1.17(0.94–1.44)	0.155

CI: confidence interval; HWE: Hardy–Weinberg equilibrium; MAF: minor allele frequency; OR: odds ratio; SNP: single nucleotide polymorphism

*Sites with HWE (*p* < 0.05) are excluded

*P*^*a*^ values is calculated with two–sided χ2

*p^*a*^ < 0.05 indicates statistical significance

**Table 3:** Association between SNPs genotypes and RA risk

SNP	Model	Genotype	Cases	Controls	OR (95%CI)	*P*
rs2050568	Dominant	C/C	184	184	1	
*FCRL1*		C/T –T/T	322	325	0.99 (0.77–1.28)	0.945
	Recessive	C/C – C/T	436	441	1	
		T/T	70	68	1.40 (0.99–1.98)	0.060
	Log-additive				1.09 (0.91–1.31)	0.348
rs2317230	Dominant	G/G	197	202	1	
*FCRL3*		G/T - T/T	309	307	1.03 (0.80–1.33)	0.806
	Recessive	G/G - G/T	419	443	1	
		T/T	87	66	1.04 (0.73–1.49)	0.821
	Log-additive				1.03 (0.86–1.23)	0.771
rs58240276	Dominant	C/C	300	325	1	
*FCRL6*		C/T –T/T	206	184	1.21 (0.94–1.56)	0.137
	Recessive	C/C – C/T	482	488	1	
		T/T	24	21	1.16 (0.63–2.11)	0.636
	Log-additive				1.17(0.94–1.45)	0.155

CI: confidence interval; OR, odds ratio; SNP: single nucleotide polymorphism

*p < 0.05 indicates statistical significance

### Stratification analysis on the association between SNPs and RA risk

When the stratification analysis by age was conducted, the results suggested that the C/T – T/T genotype of *FCRL6* rs58240276 polymorphism was significant increased the risk of RA in the old group at age >  54 years in under the dominant model (OR = 1.54, 95% CI = 1.08–2.19, *p* = 0.016). We also observed that *FCRL6* rs58240276 polymorphism was associated with an increased improved the risk of RA in the old group at age > 54 years in the log-additive model (OR = 1.41, 95% CI = 1.05–1.89, *p* = 0.021). However, no significant differences were found between *FCRL1* rs2050568 and *FCRL3* rs2317230 polymorphism and RA risk under the stratification analysis among three genetic models. The results are presented in Table 4.

**Table 4:** Association between SNPs and RA risk were stratified for age

SNP	Model	Genotype	≤ 54		> 54	
			OR (95% CI)	*P*	OR (95% CI)	*P*
rs2050568	Dominant	C/C	1		1	
*FCRL1*		C/T –T/T	0.90 (0.62–1.31)	0.592	1.08 (0.76–1.54)	0.656
	Recessive	C/C – C/T	1		1	
		T/T	1.42 (0.87–2.34)	0.165	1.43 (0.87–2.34)	0.154
	Log-additive		1.05 (0.81–1.37)	0.715	1.14 (0.89–1.47)	0.296
rs2317230	Dominant	G/G	1			
*FCRL3*		G/T - T/T	1.10 (0.76–1.59)	0.605	0.98 (0.69–1.40)	0.929
	Recessive	G/G - G/T	1			
		T/T	0.85 (0.50–1.46)	0.561	1.26 (0.77–2.05)	0.360
	Log-additive		1.01 (0.77–1.33)	0.930	1.05 (0.82–1.35)	0.689
rs58240276	Dominant	C/C	1		1	
*FCRL6*		C/T –T/T	0.93 (0.64–1.34)	0.697	**1.54 (1.08–2.19)**	**0.016**
	Recessive	C/C – C/T	1		1	
		T/T	0.80 (0.29–2.18)	0.662	1.43 (0.67–3.06)	0.355
	Log-additive		0.92 (0.67–1.28)	0.631	**1.41 (1.05–1.89)**	**0.021**

CI: confidence interval; OR: odds ratio; SNP: single nucleotide polymorphism

*p < 0.05 indicates statistical significance

Subsequently, stratification analysis by gender was performed. Our results confirmed that *FCRL1* rs2050568 T/T genotype enhanced the risk of RA in females under the recessive model (OR = 1.64, 95% CI = 1.10–2.45, *p* = 0.014). On the contrary, the G/T - T/T genotype of *FCRL3* rs2317230 was correlated with decreased RA risk in males in the dominant model (OR = 0.56 95% CI = 0.34–0.92, *p* = 0.021). For *FCRL6* rs58240276, we not observed that any association between the SNP polymorphism and RA risk in gender stratification (Table 5**)**.

**Table 5:** Association between SNPs and RA risk were stratified for gender

SNP	Model	Genotype	Male		Female	
			OR (95% CI)	*P*	OR (95% CI)	*P*
rs2050568	Dominant	C/C	1		1	
*FCRL1*		C/T –T/T	0.89 (0.54–1.46)	0.648	1.03 (0.76–1.39)	0.842
	Recessive	C/C – C/T	1		1	
		T/T	0.80 (0.38–1.69)	0.564	**1.64 (1.10–2.45)**	**0.014**
	Log-additive		0.89 (0.62–1.28)	0.533	1.17(0.95–1.43)	0.150
rs2317230	Dominant	G/G	1			
*FCRL3*		G/T - T/T	**0.56 (0.34–0.92)**	**0.021**	1.29 (0.96–1.73)	0.092
	Recessive	G/G - G/T	1			
		T/T	0.85 (0.38–1.87)	0.678	1.10 (0.74–1.65)	0.636
	Log-additive		0.69 (0.47–1.00)	0.051	1.16 (0.95–1.43)	0.153
rs58240276	Dominant	C/C	1		1	
*FCRL6*		C/T –T/T	1.30 (0.80–2.11)	0.292	1.18 (0.88–1.59)	0.274
	Recessive	C/C – C/T	1		1	
		T/T	1.35 (0.45–3.99)	0.593	1.08 (0.53–2.22)	0.835
	Log-additive		1.25 (0.83–1.87)	0.282	1.14 (0.88–1.47)	0.317

CI: confidence interval; OR: odds ratio; SNP: single nucleotide polymorphism

*p < 0.05 indicates statistical significance

### SNP - SNP interactions using MDR analysis with RA risk

**Fig. 1:**
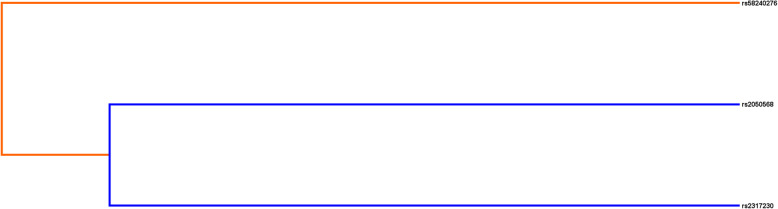
SNP-SNP Interaction Dendrogram

**Fig. 2:**
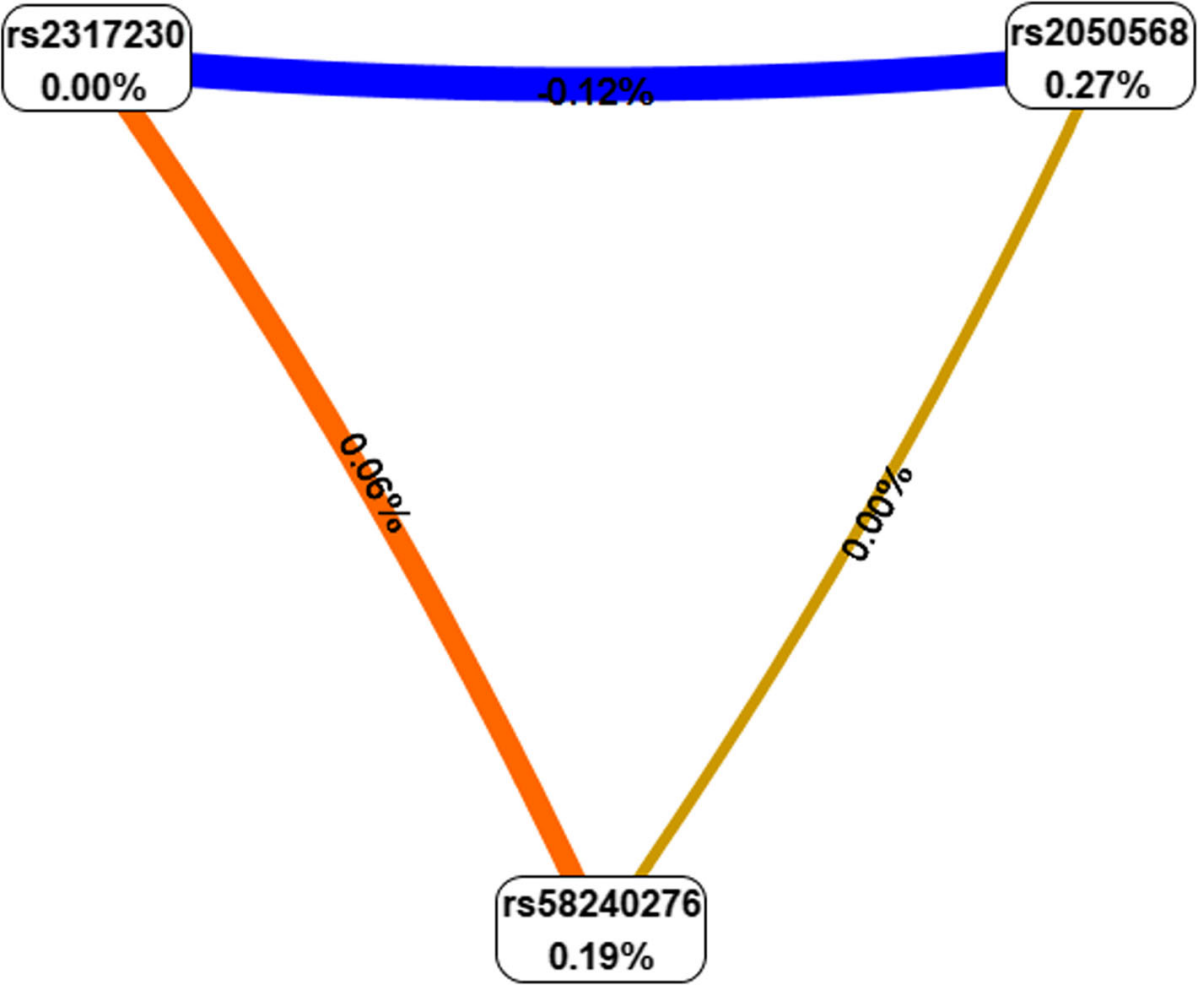
Fruchterman-Reingold, Describing Interactions Between the Three Genetic Variants

The MDR method was conducted to analyze SNP - SNP interaction. We used MDR analysis to assess the impact of the SNP - SNP interaction between the three selected SNPs in the *FCRL* (Table 6). The higher the “CV Consistency” and “Accuracy” values, the greater the interaction between SNPs. We found three models in total and found that *FCRL1* rs2050568-TT, *FCRL3* rs2317230-TT, and *FCRL6* rs58240276-TT are the best models for multi - site MDR analysis (CV consistency = 10/10, accuracy = 0.541, *p* < 0.01). In addition, models with *FCRL1* rs2050568 and *FCRL6* rs58240276 increase the risk of RA. Next, we used the interaction dendrogram of the entire genotype data set to show the SNP - SNP interaction between these two genes. As shown in Fig. 1, the bluer the color, the stronger the redundancy between SNPs. Conversely, the redder the color, the stronger the synergy between the sites. And in the risk analysis of RA, it can be observed that there is a strong redundancy between rs2050568 and rs2317230. It can be seen from Fig. 2 that there is a synergy between rs58240276 and rs2317230.

**Table 6:** MDR analysis of SNP-SNP interactions in *FCRL* gene

Model	Training Bal. Acc.	Testing Bal. Acc.	CV Consistency	Accuracy	Sensitivity	Specificity	OR (95%CI)	*p*
rs58240276	0.527	0.492	6/10	0.525	0.408	0.641	1.23 (0.96–1.59)	0.11
rs2050568,rs58240276	0.539	0.514	10/10	0.538	0.363	0.712	1.41 (1.08–1.83)	0.01*
rs2050568,rs2317230,rs58240276	0.545	0.490	10/10	0.541	0.391	0.692	1.44 (1.11–1.87)	0.0057*

Abbreviation: MDR: multifactor dimensionality reduction; SNP: single nucleotide polymorphism; CV: cross-validation; OR: odds ratio; CI: confidence interval

Note: *p* is calculated using χ^2^ test. All *p-*values in this study are two-tailed. Bold values mean statistical significance.*p < 0.05 indicates statistical significance

## Discussion

RA is a most typical autoimmune disease, and affected by various genetic and environmental factors. Multiples studies revealed that genetic factors can lead to the risk of RA and various SNPs, which have been confirmed to be associated with the susceptibility of RA [21]. Up to now, the contribution of the SNPs such as *FCRL1* rs2050568, *FCRL3* rs2317230, and *FCRL6* rs58240276 to RA risk remained unclear. In the current study, we performed a case-control study to clarify the relationship between *FCRL1* rs2050568, *FCRL3* rs2317230, *FCRL6* rs58240276 polymorphisms and RA susceptibility in the Chinese Han population. Eventually, our results suggested that these SNPs were associated with the risk of RA in different stratification by age and gender.

The *FCRL* family gene is located in 1p21–23 region, and may play a crucial role in regulation of the B cell signaling [22]. Mutations in *FCRL* gene have been reported to be correlated with numerous human diseases, including grave disease and immune system disease [23–25]. A study reported that the expressions of activating or inhibitory *FCRL1, 2,* and 4 revealed alterations in graves’ disease patients compared to healthy subjects [26]. Recently, a meta-analysis which including 916 patients with RA and 3746 healthy controls was performed, the results suggested that *FCRL3* rs17727339 showed significant correlations with RA risk [16]. In addition, Kochi et al. reported that *FCRL3* variant (−169C) was associated with RA risk in the Japanese population [24]. Whereas, a subsequent research suggested that this association was not reproduced among a Spanish and numerous US-based RA case-control subjects [25, 27]. There are differences in the incidence of *FCRL* gene polymorphisms MAF and RA between the Chinese Han population and the other populations. Ramírez-Bello et al. explored the relationship between *FCRL3* polymorphism and juvenile rheumatoid arthritis (JRA) in the Mexican population and found that *FCRL3_3* (rs7528684) and *FCRL3_6* (rs3761959) showed significant MAF difference (*p* = 0.03 and *p* = 0.01, respectively). The protective effect of *FCRL3* gene SNP on JRA disease in Mexican male patients [28]. There is a significant difference in the *FCRL3* -69 T > C (rs7528684) in the Chinese population (*p* = 0.003), and the MAF of 1381G > A (rs3761959) is not significantly different (*p* = 0.493). The *FCRL3*-169 T/C variant and the RA in the Chinese Han population is significantly related to an increased risk [29]. In our study, our study confirmed that the allele and genotype frequencies of *FCRL1* rs2050568, *FCRL3* rs2317230 and *FCRL6* rs58240276 were not interacted with RA risk in the Chinese Han population. Subsequently, we performed stratification analysis by age and gender. The results demonstrated that the C/T – T/T genotype of *FCRL6* rs58240276 polymorphism was increased the risk of RA in age > 54 years (*p* = 0.016). During the same time, we also confirmed that *FCRL1* rs2050568 T/T genotype enhanced the risk of RA in females (*p* = 0.014). In contrast, the G/T - T/T genotype of *FCRL3* rs2317230 was correlated with a decreased RA risk in males (*p* = 0.021).

Various limitations need to be considered in our study, such as the information of samples was little. All in all, we demonstrated that *FCRL1* rs2050568 T/T genotype, *FCRL3* rs2317230 G/T - T/T genotype, and *FCRL6* rs58240276 T/T genotype were associated with RA risk in Chinese Han people, when the stratified analysis by age and gender was performed. Our study provided a new insight into the pathogenesis of this disease. In the future, the detailed molecular mechanism by which the above mentioned polymorphisms influencing the occurrence and development of RA was necessary to be investigated.

## Conclusions

In a word, we firstly provided new evidence for the relationship between the selected variants and RA risk, which may support for the screening of RA in the Han Chinese population and shed light on the mechanism of RA.

## Supplementary Information

**Additional file 1: Supplementary Table 1.** False-Positive Report Probability Values for Associations Between the Risk of rheumatoid arthritis and the Frequency of Variants and Model of *FCRL* Gene in the Chinese Han population.

## Data Availability

All data generated or analysed during this study are included in this published article.

## Abbreviations

RA: Rheumatoid arthritis
SNP: Single nucleotide polymorphism
OR: Odds ratio
CI: Confidence interval
MDR: Multifactor Dimensionality Reduction
GWAS: Genome-wide association study
MAF: Minor allele frequency
EDTA: Ethylenediamine tetraacetic acid
HWE: Hardy–Weinberg equilibrium
LD: Linkage disequilibrium
