# Supplementary material for: Fc receptor-like 1, 3, and 6 variants are associated with rheumatoid arthritis risk in the Chinese Han population
Source: Genes Environ. 2021 Oct 7;43:42. doi: 10.1186/s41021-021-00213-2 (PMC8499487; doi:10.1186/s41021-021-00213-2)
Supplement: Supplementary file 1 — Additional file 1: Supplementary Table 1. False-Positive Report Probability Values for Associations Between the Risk of rheumatoid arthritis and the Frequency of Variants and Model of FCRL Gene in the Chinese Han population. [file 41021_2021_213_MOESM1_ESM.docx]

Supplementary table 1. False-Positive Report Probability Values for Associations Between the Risk of rheumatoid arthritis and the Frequency of Variants and Model of *FCRL* Gene in the Chinese Han population.

| Positive results in this study | Variants/Model | Crude OR (95% CI) | P-value^a^ | Statistical power^b^ | Prior probability | | | | |  |
| --- | --- | --- | --- | --- | --- | --- | --- | --- | --- | --- |
|  |  |  |  |  | 0.25 | 0.1 | 0.01 | 0.001 | 0.0001 | |
| The association analysis stratified by age between *FCRL6* rs58240276 polymorphisms and RA risk among RA patients > 54 years. | C/T –T/Tvs.CC | 1.54(1.08-2.19) | 0.016 | 0.927 | **0.050** | **0.136** | 0.634 | 0.946 | 0.994 | |
|  | Log-additive | 1.41(1.05-1.89) | 0.021 | 0.990 | **0.061** | **0.164** | 0.683 | 0.956 | 0.995 | |
| In female RA patients, the correlation analysis between *FCRL1* rs2050568 polymorphism and rheumatoid arthritis risk. | TT vs.C/C – C/T | 1.64(1.10-2.45） | 0.014 | 0.834 | **0.054** | **0.145** | 0.651 | 0.950 | 0.995 | |
| In male RA patients, the correlation analysis between *FCRL3* rs2317230 polymorphism and rheumatoid arthritis risk. | G/T - T/T vs.GG | 0.56(0.34-0.92） | 0.021 | 0.673 | **0.090** | 0.228 | 0.765 | 0.970 | 0.997 | |

Abbreviation: CI, confidence interval; OR, odds ratio;

^a^Chi-square test was used to calculate the variants and model frequency distributions.

^b^Statistical power was calculated using the number of observations in the subgroup and the OR and P values in this table.
